# Facile synthesis of a hollow Ni–Fe–B nanochain and its enhanced catalytic activity for hydrogen generation from NaBH_4_ hydrolysis†

**DOI:** 10.1039/c8ra03848a

**Published:** 2018-07-19

**Authors:** Jie Guo, Yongjiang Hou, Bo Li

**Affiliations:** School of Environmental Science and Engineering, Hebei University of Science and Technology Shi Jiazhuang 050018 China houyongjiang122@163.com

## Abstract

A hollow Ni–Fe–B nanochain is successfully synthesized by a galvanic replacement method using a Fe–B nanocomposite and a NiCl_2_ solution as the template and additional reagent, respectively. Both the concentration of Ni and the morphology of the resulting Ni–Fe–B alloy are controlled by varying the duration of the replacement process during the synthesis. The Ni–Fe–B sample synthesized for 60 min (Ni–Fe–B-60) shows the best catalytic activity at 313 K, with a hydrogen production rate of 4320 mL min^−1^ g_cat_^−1^ and an activation energy for the NaBH_4_ hydrolysis reaction of 33.7 kJ mol^−1^. The good performance of Ni–Fe–B-60 towards the hydrolysis of NaBH_4_ can be ascribed to both hollow nanochain structural and electronic effects. Furthermore, the effects of temperature, catalyst amount, and concentration of NaOH and NaBH_4_ on the hydrolysis process are systematically studied, and an overall kinetic rate equation is obtained. The hollow Ni–Fe–B nanochain catalyst also shows good reusability characteristics and maintained its initial activity after 5 consecutive cycles.

## Abstract 2

A hollow Ni–Fe–B nanochain is successfully synthesized by a galvanic replacement method using a Fe–B nanocomposite and a NiCl_2_ solution as the template and additional reagent, respectively.

## Graphical abstract

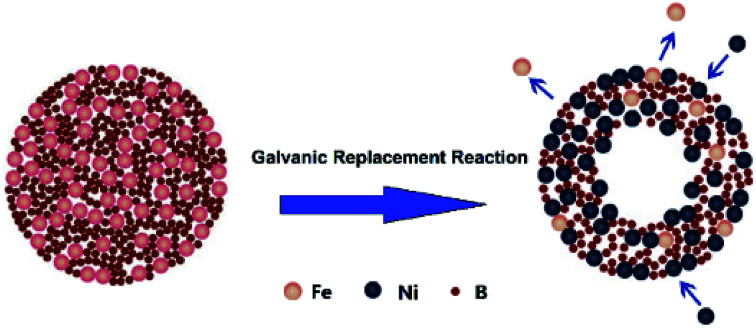

## 1. Introduction

In recent years, hydrogen has come to be widely accepted as a source of clean energy and possible replacement for fossil fuels, which are responsible for smog, acid rain, and greenhouse effect issues.^1^ Unlike fossil fuels, the only combustion product of hydrogen is water, which is very gentle on the environment.^2,3^ However, storage and transportation of hydrogen have become crucial concerns in the development of hydrogen energy technologies. In recent years, chemical borohydride materials, such as LiBH_4_, NH_3_BH_3_, and NaBH_4_, which have high hydrogen density and low molecular weight, have drawn considerable interest as promising hydrogen storage materials.^4–6^ Among the chemical borohydrides in contention, NaBH_4_ is considered the most promising source of hydrogen owing to its stability in alkaline solutions, easily controllable hydrogen generation rate (HGR), moderate reaction temperature, and nontoxic hydrolytic byproducts.^7–9^ NaBH_4_ releases 4 mol of H_2_ in the presence of a catalyst, as shown in eqn (1):^3,10^

1 NaBH_4_ + (2 + *x*)H_2_O → NaBO_2_·*x*H_2_O + 4H_2_ + Heat

A large amount of work has focused on the research and development of the appropriate catalyst to be used. Noble metal (*e.g.*, Pt, Ru, and Pd) materials^11–17^ have been reported to promote high catalytic activity for the hydrolysis of NaBH_4_. However, they have many limitations in real applications due to their high costs and short lifetimes. In this sense, catalysts based on cheap transition metals such as Fe, Co, Ni, and Cu have attracted significant attention.^18–29^ Moreover, it is necessary to study the preparation methods of non-noble metal catalysts with high efficiency. The catalysts generally synthesized through traditional chemical reduction of metal ions with borohydride (BH_4_^−^) would have limited catalytic activity resulting from agglomeration and low surface area issues, poor electronic conductivity (for e^−^ transportation), and difficult separation from the reaction media after the reaction. To further improve catalytic activity, these problems must be addressed. One strategy is to reduce the catalyst particle size to increase surface area. The synthetic method involves the chemical reduction of a nickel complex precursor (such as hydrazine, ethylenediamine) while controlling the rate of the exothermic reaction to form fine small-sized particles.^30–32^ However, the smaller the nanoparticle, the harder it is to avoid agglomeration. Another method is to synthesize bimetallic catalysts and adjust the optimal molar ratio of two metals. More recently, Nie *et al.* found that the Ni–Fe–B catalysts have an enhanced catalytic activity with respect to Ni–B powders.^33^ The synergetic effect of the most suitable ratio of two metal atoms can facilitate hydrolysis. However, the degree of mixing bimetallic catalysts synthesized by chemical reduction methods is limited in their ability to reach the atomic cluster level. Thus, adjusting the distribution and dispersion of metal atoms is not possible, which could weaken the synergetic effect.

**Fig. 1:**
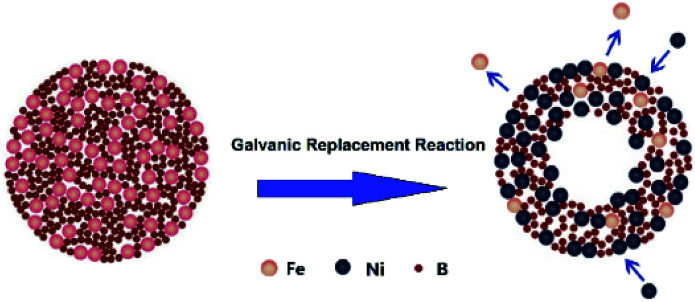
Formation mechanism of hollow Ni–Fe–B sample.

High surface area hollow nanostructure catalysts with well-controlled properties can be facile prepared using the galvanic replacement method.^34^ Currently, this technique has been used for preparing binary noble metal catalysts^35,36^ or combinations of transition and noble metals.^37,38^ In this context, a hollow Ni–Fe–B catalyst for NaBH_4_ hydrolysis was first synthesized by galvanic replacement reaction. The synthetic mechanism was illustrated in Fig. 1.

Fe–B nanocomposites were used as templates and a NiCl_2_ solution served as an additive. Upon addition of an aqueous NiCl_2_ solution to an aqueous suspension of Fe–B nanocomposites, the galvanic replacement started immediately at the site with the high surface energy. Fe atoms were then oxidized and incorporated into the solution. Simultaneously, the electrons quickly migrate to the surface of the nanoparticle and become captured by Ni^2+^ ions, generating Ni atoms by reduction. The newly formed Ni atoms tend to deposit epitaxial on the surface of the Fe–B nanocomposite. The deposition of Ni was accompanied by the formation of a homogenous alloy of Fe with the Ni on surface, which is more thermodynamically stable than a mixture of segregated Ni and Fe. Continuous dissolution of Fe from the template resulted in the transformation of the nanoparticle into a nanostructure characterized by a hollow interior and an alloyed shell. The obtained hollow Ni–Fe–B was tested for hydrogen generation from the hydrolysis of NaBH_4_. It exhibited high surface area and excellent catalytic activity for the hydrolysis reaction.

## 2. Material and methods

### 2.1 Catalyst preparation

In a typical procedure, Fe–B nanocomposite was used as the template, which was prepared by reducing a 0.5 M ferric chloride [FeCl_3_·6H_2_O] (Aladdin Reagent Co., Shanghai, China) and 0.5 M tartaric acid (Aladdin Reagent Co., Shanghai, China) mixed solution (10 mL each) with potassium borohydride (Chuandong Chemical Co., Ltd., Chongqing, China). The products were then filtered and washed several times with deionized water and ethanol. For the synthesis of Ni–Fe–B samples by the galvanic replacement reaction, the Fe–B template was mixed in 10 mL of 0.5 M nickel chloride [NiCl_2_·6H_2_O] (Aladdin Reagent Co., Shanghai, China) solution and sonicated for 30, 60 and 120 min, which were aliquoted out and marked as Ni–Fe–B-30, Ni–Fe–B-60 and Ni–Fe–B-120, respectively. Finally, the as-obtained Ni–Fe–B samples were then filtered, washed several times with deionized water and ethanol, and vacuum dried at 330 K. A Ni–Fe–B sample denoted as Ni–Fe–B-c was prepared by traditional chemical reduction, produced by reducing a 0.5 M ferric chloride [FeCl_3_·6H_2_O] and 0.5 M nickel chloride [NiCl_2_·6H_2_O] aqueous mixed solution (10 mL each) with potassium borohydride. The Ni–B sample was prepared by reducing 10 mL of a 0.5 M nickel chloride [NiCl_2_·6H_2_O] aqueous solution with potassium borohydride.

### 2.2 Catalyst characterization

The morphologies of the Ni–Fe–B samples were characterized by transmission electron microscopy (TEM, JEOL, JEM-2100). The quantitative chemical compositions of catalysts were determined with energy-dispersive X-ray spectroscopy (EDX, Genesis Spectrum, 200 kV). The structures of the catalysts were analyzed by X-ray diffraction (XRD, Rigaku, D/max 2500PC) with Cu Kα radiation (*γ* = 1.5418 Å) in the 2*θ* range of 10–80. X-ray photoelectron spectroscopy (XPS, Perkin, PHI-1600 ESCA) measurements were recorded with a spectrophotometer using a Mg X-ray (*hν* = 1253.6 eV) source for excitation; the binding energy (BE) values were calibrated using C 1s = 284.6 eV as a reference. Hydrogen temperature-programmed desorption (H_2_-TPD) measurements were performed on a TP-5076 instrument (Tianjin Xianquan Instrument Co. Ltd., China). The BET surface area was measured using a surface area analyzer (Quantachrome Instruments, Autosord-IQ).

### 2.3 Hydrolysis of NaBH_4_ measurements

Water displacement method was used to measure the rate of hydrogen generation.^8,9^ The reaction was carried out in a glass reactor equipped with thermostatic bath. Then, a flask filled with water was connected to the reaction chamber to measure the volume of hydrogen gas to be evolved from the reaction. Typically, the hydrolytic dehydrogenation of NaBH_4_ was determined at 313 K. NaBH_4_ (1 wt%) and NaOH (2 wt%) were mixed in a reactor containing 10 mL of water and 10 mg of catalyst. The reaction was started by closing the reactor. The volume of hydrogen gas evolved was measured by recording the displacement of the reactor's water level. During the reaction process, a wet gas meter was used to measure the cumulative volume of the hydrogen generation rate (mL min^−1^). The experiments were performed using a stirring speed of 500 rpm to exclude external mass-transfer limitations. To study the effect of the reaction temperature on the reaction rate, NaBH_4_ hydrolysis was performed at different temperatures (303, 308, 313, and 318 K).

## 3. Results and discussion

### 3.1 Characterization of catalysts

**Fig. 2:**
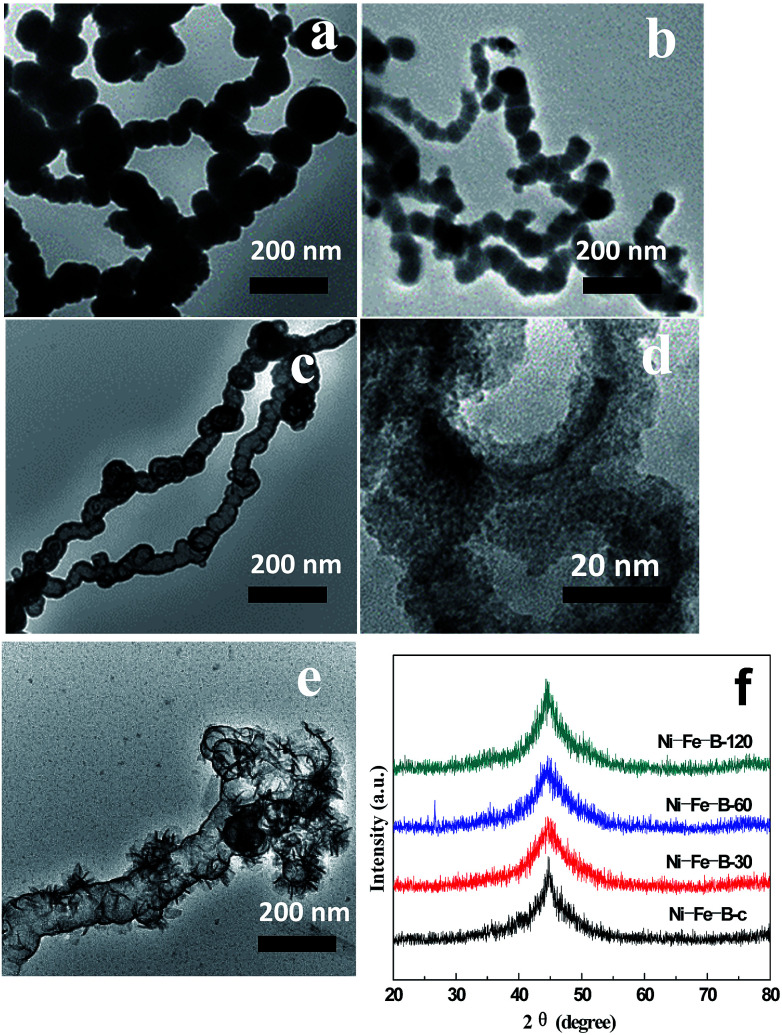
TEM images of (a) Ni–Fe–B-c, (b) Ni–Fe–B-30, (c) Ni–Fe–B-60, (d) Ni–Fe–B-60 at higher magnification, (e) Ni–Fe–B-120, and (f) XRD patterns of Ni–Fe–B samples.

Fig. 2 shows TEM micrographs of the Ni–Fe–B samples. As shown in Fig. 2a, the Ni–Fe–B-c sample spontaneously formed chain structures because of their magnetism. The specific surface area of the Ni–Fe–B-c sample was 52.9 m^2^ g^−1^.

A highly energetic galvanic replacement reaction took place immediately upon the addition of an aqueous NiCl_2_ solution to the aqueous suspension of Fe–B nanochains, which is based on the reduction potentials^39^ according to the following equations:

2 Ni^2+^_(aq)_ + 2e^−^ → Ni_(s)_, −0.25 V *vs.* SHE

3 Fe_(s)_ → Fe^2+^_(aq)_ + 2e^+^, −0.44 V *vs.* SHE

4 Fe_(s)_ + Ni^2+^_(aq)_ → Fe^2+^_(aq)_ + Ni

As a result, Fe atoms were oxidized and incorporated into the solution, leading to the formation of loose Ni–Fe–B-30 in the sample. Simultaneously, the electrons migrate quickly to the surface of the nanoparticle and were captured by Ni^2+^ ions, generating Ni atoms on the Fe–B surface *via* reduction. The specific surface area of the Ni–Fe–B-30 sample was 85.5 m^2^ g^−1^ (Fig. 2b). Further dealloying (Ni–Fe–B-60) resulted in larger holes, producing hollow nanochain showing (Fig. 2c and d) a maximum specific surface area of 118.6 m^2^ g^−1^. As shown in Fig. 2e, Ni–Fe–B-120 showed disintegrated hollow structures which were subsequently reassembled, resulting in a material with less surface area (74.8 m^2^ g^−1^). By increasing the replacement reaction time, the structure of the Ni–Fe–B samples became increasingly loose, producing materials with higher specific surface areas. After 120 min of reaction, the hollow nanochain structures disappeared, decreasing the specific surface areas. Fig. 2f shows the XRD spectra of the Ni–Fe–B samples. Only one broad peak, at a 2q of 45°, was observed, this pattern being characteristic of anamorphous structure and in line with previous results on Ni–Fe–B amorphous alloys.^16^

**Fig. 3:**
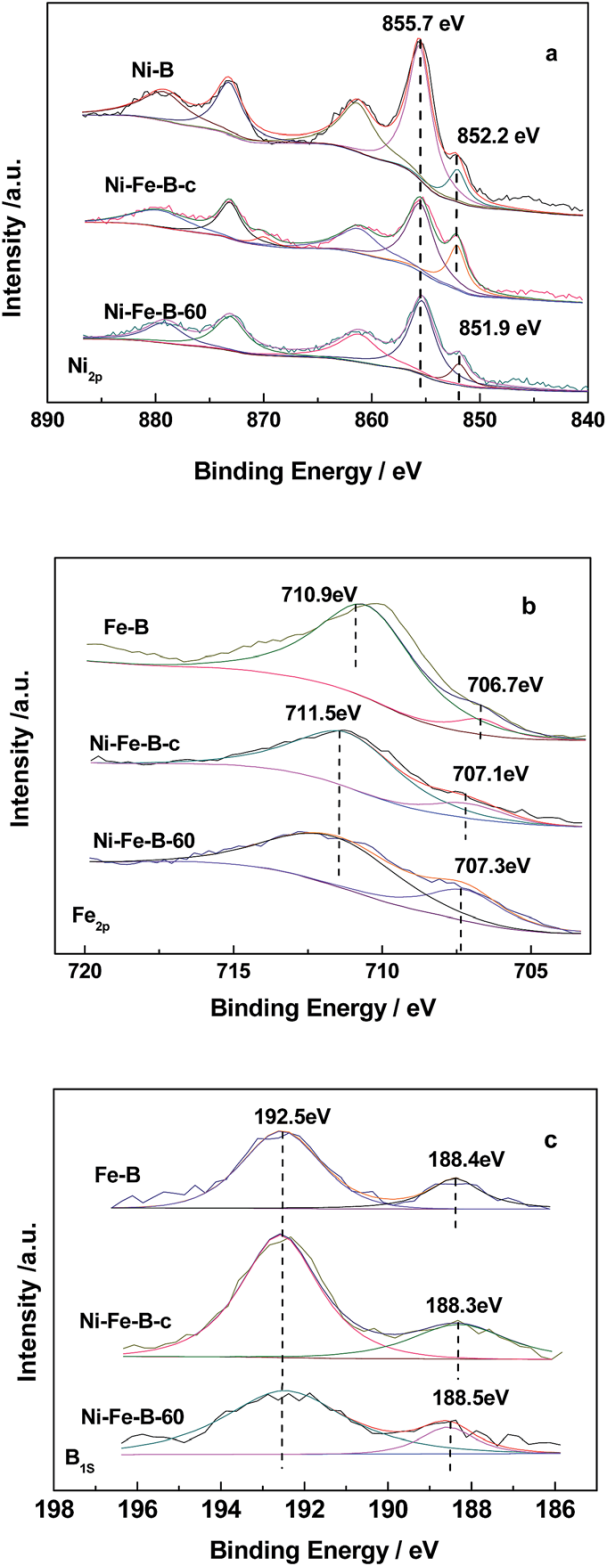
XPS spectra of Ni_2p_ (a), Fe_2p_ (b) and B_1s_ (c) state.

Fig. 3 shows the XPS analysis of the catalysts. Fig. 3a shows the Ni spectra of the catalysts, with two peaks in the Ni_2p_3/2__ level being observed for Fe–B, Ni–B, Ni–Fe–B-c, and Ni–Fe–B-60. These results indicated that Ni was present in both elemental and oxidized states, the latter of which resulted from partial oxidation reactions during sample preparation before XPS measurements.^40^ The binding energy (BE) of the elemental nickel for both of Ni–B and Ni–Fe–B-c catalysts were 852.2 eV. Compared to the BE of the elemental nickel with that of Ni–B and Ni–Fe–B-c catalysts, there is negative shift of 0.3 eV for the Ni–Fe–B-60 (851.9 eV). Two peaks were also observed for the Fe_2p3/2_ level in the Fe–B, Ni–Fe–B-c, and Ni–Fe–B-60 samples (Fig. 3b). In the case of Fe–B, the peaks at BE values of 706.7 and 710.9 eV were representative of elemental and oxidized iron species, respectively. These two peaks were shifted to higher BE values (by 0.4 and 0.6 eV) for the Ni–Fe–B-c sample as compared to that of Fe–B, which are related to the electron transfer from Fe to Ni. In the case of the Ni–Fe–B-60 sample, these values were both shifted to higher values by 0.6 eV. As shown in Fig. 3c, two peaks were observed for the B_1s_ level in all the cases and ascribed to elemental and oxidized boron species. The BE values of elemental B in Fe–B (188.4 eV), Ni–Fe–B-c (188.3 eV), and Ni–Fe–B-60 (188.5 eV) were shifted to higher values than pure amorphous B (187.1 eV),^30^ indicating some electron transfer from the alloyed B to the metal (Ni or Fe) in all samples. The BE values of elemental B and Fe for the Ni–Fe–B-60 sample were higher than those of Ni–Fe–B-c. These results suggested that more electrons transfer from B and Fe to Ni in the Ni–Fe–B-60 sample, which caused the BE of the elemental nickel to undergo a negative shift. Specifically, there were more electron-enriched Ni active sites on the surfaces of the Ni–Fe–B-60 sample.

**Fig. 4:**
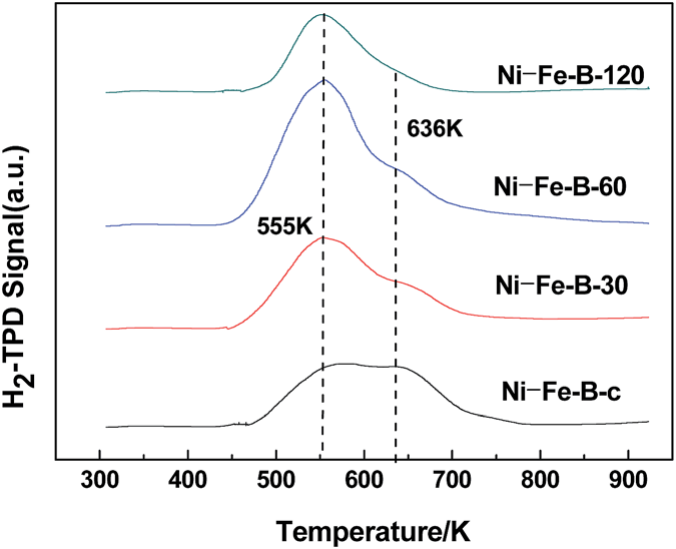
H_2_-TPD images of Ni–Fe–B samples.

Fig. 4 shows the H_2_-TPD spectra exhibited two peaks at 555 K and 636 K in all cases, indicating the presence of two adsorption sites on the surface of the samples. The area of the peak at higher temperature decreased significantly with the duration of the replacement reaction. The active sites on the surface of the catalysts tended to be uniform. The Ni–Fe–B-60 sample showed a significantly larger peak area at 555 K compared to the other peak, revealing more uniform distribution of Ni active sites compared to the rest of the Ni–Fe–B samples.

### 3.2 Catalytic hydrolysis reaction of sodium borohydride

**Fig. 5:**
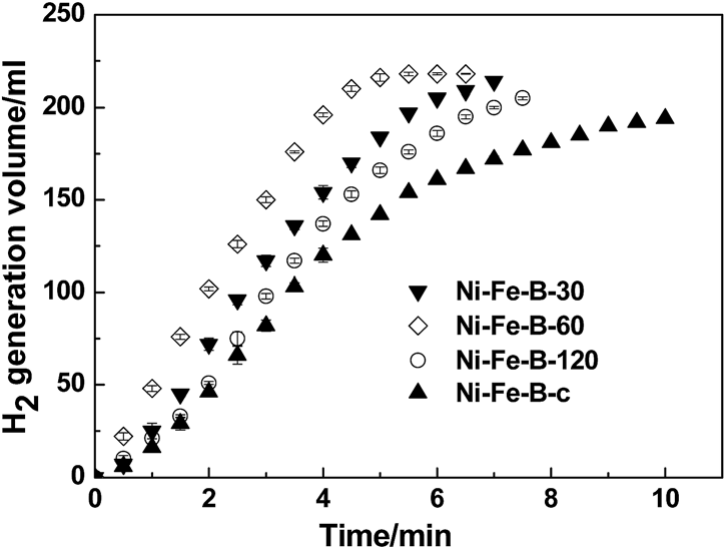
Catalytic hydrogen generation from the hydrolysis of mix solution of 1wt% of NaBH_4_ + 2 wt% of NaOH at 313 K and 10 mg catalyst, comparative study of four catalysts (i) Ni–Fe–B-c, (ii) Ni–Fe–B-30, (iii) Ni–Fe–B-60 and (iv) Ni–Fe–B-120.

The above characterizations imply that the as-synthesized Ni–Fe–B-60 sample may be catalytically active for the NaBH_4_ hydrolysis reaction. Therefore, we further compared the catalytic properties of four different catalysts (Ni–Fe–B-c, Ni–Fe–B-30, Ni–Fe–B-60, and Ni–Fe–B-120 at 313 K) in Fig. 5. The H_2_ generation rate of all catalysts first increased until reaching a maximum, then decreased, revealing an order of the reaction kinetic different than zero. Ni–Fe–B-60 showed the best catalytic performance towards the NaBH_4_ hydrolysis reaction, with a maximum hydrogen generation rate of 4320 mL min^−1^ g_cat_^−1^. The hydrogen generation rates for the rest of the catalysts are presented in Table 1.

**Table 1:** Maximum hydrogen generation rate obtained from the hydrolysis of alkaline NaBH_4_^a^

Catalyst	Maximum hydrogen generation rate (mL min^−1^ g^−1^ catalyst)
Ni–Fe–B-c	1940
Ni–Fe–B-30	2734
Ni–Fe–B-60	4320
Ni–Fe–B-120	3058

a Hydrolysis was carried out using NaBH_4_ (1 wt%) and NaOH (2 wt%) solution by 10 mg catalyst.

The high catalytic activity of Ni–Fe–B-60 can be explained by three main factors: (i) the hollow structure of the Ni–Fe–B-60 nanochain, (ii) enhanced adsorption of BH_4_^−^ on electron-enriched Ni active sites, and (iii) hydrogen spillover on Fe. A hollow structure with a larger specific surface area facilitated the catalytic reaction. Most of the Ni generated by a galvanic replacement that avoided agglomeration was on the Fe–B surface and completely exposed for catalytic reaction, as presented in Fig. 1 and 2. Fe showed low catalytic activity for the hydrolysis of the NaBH_4_ solution.^21^ The active sites in the Ni–Fe–B catalysts are Ni. The large number of uniform electron-enriched Ni active sites deposited on the surface of Ni–Fe–B-60 can be explained by the electron transfer from B and Fe to Ni, as analysis in XPS and H_2_-TPD.

Currently, it is generally accepted that metal (M)-catalyzed hydrolysis of NaBH_4_ involves the dissociative chemisorption of BH_4_^−^ on the catalyst surface as the first kinetic step.^41–45^ Because of this, our experiments showed enhanced adsorption of the BH_4_^−^ species on electron-enriched Ni active sites on the Ni–Fe–B-60 surface. BH_4_^−^ then dissociates to form Ni–BH_3_^−^ and Ni–H intermediates. According to Holbrook and Twist,^46^ Ni–BH_3_^−^ subsequently reacts with H_2_O, possibly *via* a BH_3_ intermediate, to generate another Ni–H species and BH_3_(OH)^−^, as presented in eqn (5)–(8).

5 2Ni + BH_4_^−^ ⇄ Ni–BH_3_^−^ + Ni–H

6 Ni–BH_3_^−^ ⇄ BH_3_ + Ni×e^−^

7 Ni×e^−^ + H_2_O → Ni–H + OH^−^

8 BH_3_ + OH^−^ → BH_3_(OH)^−^

The former undergoes stepwise replacement of B–H bonds by B–OH^−^ bonds and finally yields Ni–B(OH)_3_^−^, as presented in eqn (9)–(11); the latter combines with two Ni–H to yield H_2_ and to regenerate the active sites, as presented in eqn (12).

9 2Ni + BH_3_(OH)^−^ → Ni–BH_2_(OH)^−^ + Ni–H

10 2Ni + BH_2_(OH)_2_^−^ → Ni–BH(OH)_2_^−^ + Ni–H

11 2Ni + BH(OH)_2_^−^ → Ni–B(OH)_3_^−^ + Ni–H

12 Ni–H + Ni–H → Ni–H_2_ + Ni

Obviously, the adsorption of H_2_ was also enhanced for electron-enriched Ni active sites. During the hydrolysis reaction, H_2_ agglomeration and coverage of the active sites could block the formation of new active sites for continuous adsorption of the BH_4_^−^ and H_2_O species. Here, Fe could prompt hydrogen spillover from the system (eqn (13) to (14)) and facilitate the formation of new active sites for adsorption of the BH_4_^−^ species, which was consistent with other studies.^36^

13 Ni–H_2_ + Fe → Fe–H_2_ + Ni

14 Fe–H_2_ → Fe + H_2_

Most importantly, the catalytic hydrolysis process involves not only surface reactions but also the diffusion and release of hydrogen in the metal catalysts. Ni–Fe–B-60 has a well-dispersed Ni atomic cluster with optimal content on the surface of Fe–B, prepared using the galvanic replacement method, which could produce a better synergetic effect than the catalysts produced by the chemical reduction of Fe and Ni ions. The Ni/(Ni + Fe) molar ratios for the Ni–Fe–B-30, Ni–Fe–B-60, and Ni–Fe–B-120 catalysts were 0.31, 0.51, and 0.78, respectively, as determined by EDX (ESI, Fig. S1†). When the content of Ni exceeded a certain amount (Ni–Fe–B-120), the synergetic effect was weakened due to the decrease of the relative amount of Fe, resulting in a decline in catalytic activity.

### 3.3 Intrinsic kinetic study

NaBH_4_ hydrolysis reactions follow very complicated mechanisms when catalyzed by heterogeneous catalysts. It is of great significance to investigate the kinetics of the NaBH_4_ hydrolysis reaction since it can provide useful information about the role of many experimental factors affecting the hydrogen generation rate.^7,22^ So, it is necessary to study the kinetic properties of NaBH_4_ hydrolyzed by Ni–Fe–B-60. During the NaBH_4_ hydrolysis, the solution temperature, the amount of catalyst, and the concentrations of NaOH and NaBH_4_ determine the reaction rate.^47^ The hydrogen generation rate equation can be expressed as:15
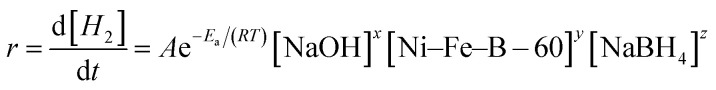
here, *r* is the hydrogen generation rate (mL min^−1^), *A* is the pre-exponential factor, *R* is the gas constant (8.3143 J mol^−1^ K^−1^), *E*_a_ is the activation energy (kJ mol^−1^), *T* is the hydrolysis temperature (K), and *x*, *y*, *z* are the reaction orders with respect to the amount of catalyst and the concentrations of NaOH and NaBH_4_, respectively.

**Fig. 6:**
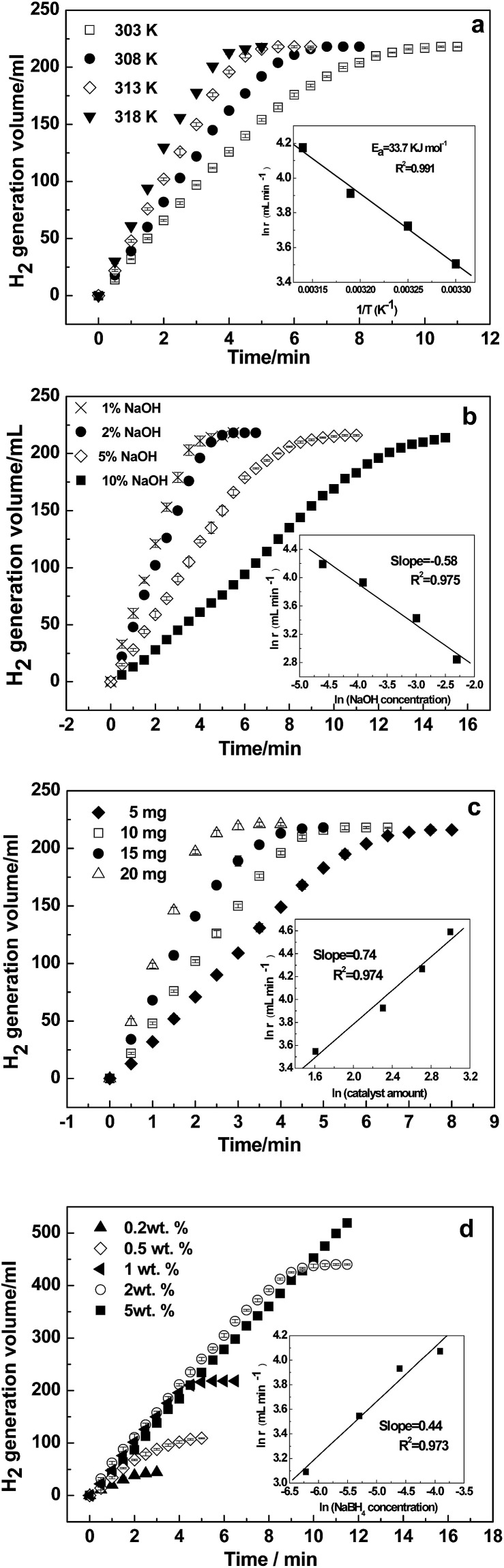
Effects of temperature (a), NaOH concentration (b), amount of catalyst (c), and NaBH_4_ concentration (d) on NaBH_4_ hydrolysis using the Ni–Fe–B-60 catalyst.

The kinetics for the hydrolysis of NaBH_4_ depends on the performance of the catalysts. Kinetic studies at different temperatures were performed using the optimized solution conditions described above (*i.e.*, 1 wt% NaBH_4_ + 2 wt% NaOH and 10 mg Ni–Fe–B-60). Fig. 6a depicts the hydrogen generation rates from 303 to 318 K. The reaction rate increased significantly with temperature. The inset Arrhenius plot of ln(*r*) is plotted against the reciprocal of the absolute temperature (1/T). From the slope of the straight line, *E*_a_ was calculated to be 33.7 kJ mol^−1^. Hydrogen generation properties for the hydrolysis of NaBH_4_ catalyzed by various reported catalysts are displayed in Table 2. As shown, our obtained *E*_a_ value was lower than that of previously reported catalysts, such as Ni–Fe–B,^33^ Co–B@Ni/RGO,^45^ and plasma treated Co–B–P,^48^ but it was higher than that of Co–Fe–B,^19^ p(AAGA)-Co,^24^ p(AAc)-Co^25^ and Cu–Co.^49^ The hydrogen generation rate of the hollow Ni–Fe–B nanochain catalyst is higher than most of the other catalysts listed in Table 2, except for Co–W–P/γ-Al_2_O_3_ (ref. 27) and Co–Ni–Mo–P/γ-Al_2_O_3_.^28^

**Table 2:** Comparison of morphology, BET, HGR, reusability and the activation energy of our catalyst and other catalysts reported in the literature^a^

Catalyst	Morphology	BET surface (m^2^ g_cat_^−1^)	Remaining activity	Activation energy (kJ mol^−1^)	Hydrogen generation rate (mL min^−1^ g_cat_^−1^)	Reference
Ni–Co/r-GO	Particle	—	53.5% after 5 cycles	55.12	1280	6
Co–Fe–B	Particle	128.3	—	29.09	4310	19
Cu–Fe–B	Nanosheet	186.7	75% after 3 cycles	57	—	22
Ni–Fe–B	Particle	—	—	57	2910	33
Co–B@Ni/RGO	Particle	—	95% after 3 cycles	44.1	—	45
Plasma treated Co–B–P	Particle	38.29	—	49.11	3976	50
Cu–Co	3D foam-like	—	∼75% after 5 cycles	13.9	3300	49
Ni–Co–B	Particle	17.07	—	—	708	54
p(HEMA)-Co	Porous	—	57.72% after 5 cycles	37.01	1596	26
p(AAGA)-Co	Porous	—	95% after 7 cycles	26.62	3019	24
Co–Ni–Mo–P/γ-Al_2_O_3_	Particle	—	80% after 5 cycles	52.43	13 842	28
Co–W–P/γ-Al_2_O_3_	Particle		67% after 6 cycles	49.58	11 820	27
Ni–Fe–B-60	Hollow nanochain	118.6	90% after 5 cycles	33.7	4320	This study

a —, Not reported or no detailed data are available.

The hydrolysis of NaBH_4_ is affected by the NaOH concentration.^22,33^ To hinder NaBH_4_ self-hydrolysis, NaOH is often added to NaBH_4_ solutions as a stabilizer. The hydrolytic reaction at ambient temperatures is greatly accelerated upon addition of catalysts. Fig. 6b shows the effects of the NaOH concentration on the reaction rate at 313 K in a 1 wt% NaBH_4_ solution and 10 mg of catalyst. The variation in NaOH was 1, 2, 5 and 10 wt%. The results show the hydrogen generation rate decreased gradually with increasing NaOH concentrations. At a low NaOH concentration of 1 wt%, the reaction rate was quite fast, and the hydrogen production process completed within 4 min. When the NaOH concentration increased to 10 wt%, the hydrogen production rate reduced drastically, extending the whole reaction process to 15 min, more than 4 times as much time as when 1 wt% NaOH was used. The ln(*r*) *versus* ln(NaOH concentration) in the inset of Fig. 6b shows that the slope of the straight line was −0.58, confirming NaOH had a negative effect on the hydrogen generation rate. This is consistent with previous reports on Ni- and Co-based catalysts.^33,50,51^ Excessive concentration of NaOH reduces the solubility of NaBO_2_, and then the active sites are blocked by the precipitation of NaBO_2_ on the surface of the catalyst. Moreover, the high viscosity and stability of NaBH_4_ at high pH values are also responsible for the observed decrease in activity.^52–54^

The effect of varying catalyst loadings (5, 10, 15 and 20 mg) on the hydrogen generation rate was determined. As shown in Fig. 6c, the hydrogen generation rate increased with the amount of Ni–Fe–B-60 catalyst, indicating that the hydrogen generation rate can be controlled by varying the catalyst loading. In addition, the ln(*r*) *versus* ln(catalyst amount) is plotted in the inset of Fig. 6c, from which we can see the ln(*r*) changed almost linearly with the ln(catalyst amount) with the slope of the straight line being 0.74.

NaBH_4_ is the hydrogen source in the hydrolysis reaction, so its concentration is the crucial factor that determines the kinetics of the reaction. Therefore, the effect of NaBH_4_ concentration on the hydrogen generation rate was further studied using NaBH_4_ at different concentrations (0.2, 0.5, 1.0, 2.0 and 5.0 wt%). Fig. 6d shows the hydrogen generation curves with the concentration of NaBH_4_ varied from 0.2 wt% to 5.0 wt%. The hydrogen generation rate increased significantly when the concentration of NaBH_4_ increased from 0.2 wt% to 2 wt%. However, when the NaBH_4_ concentration further increased to 5 wt%, the hydrogen generation rate dropped remarkably. Hence, only the experiments with NaBH_4_ concentrations less than 5 wt% were used to determine the reaction order in the inset of Fig. 6d, where the slope was 0.44. This is consistent with previous reports on Ni- or Co-based catalysts.^22,47^

According to the above investigations, the activation energy and the *x*, *y* and *z* factors for the NaBH_4_ hydrolysis reaction were obtained, and the final overall kinetic equation with the concentration of NaBH_4_ less than 10 wt% can be expressed as:16
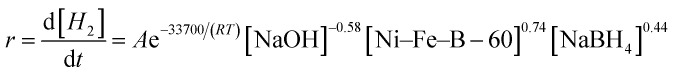


The results could provide valuable information to design a possible reactor for practical applications.

**Fig. 7:**
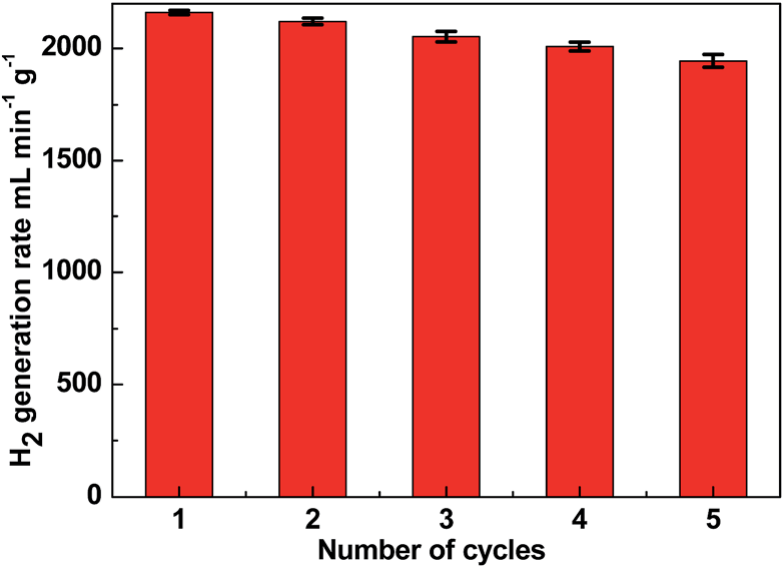
Hydrogen generation rate of Ni–Fe–B-60 reusability test of catalyst (10 mg) in successive five cycles.

One of the limiting factors for the application of the catalyst is deactivation. To investigate the stability of the catalyst, the reusability of Ni–Fe–B-60 was tested. In our study, we used 10 mg of catalyst per 1 wt% of NaBH_4_ with respect to H_2_O (10 mL, *i.e.*, 2 wt% NaOH). The experiment was conducted five times. After the reaction, the Ni–Fe–B-60 catalysts were separated by filtration. Since Fe and Ni are magnetic, the catalyst is easy to separate from the reaction solution, as illustrated by the magnetic strip in Fig. S2.† Then the catalyst was washed with distilled water and dried in a vacuum oven at 333 K before being reused for the next run. The results are depicted in Fig. 7. The catalytic activity of Ni–Fe–B-60 catalysts for the hydrolysis of NaBH_4_ did not decrease significantly after five runs, which demonstrated that the catalyst was stable in this system.

## 4. Conclusions

In summary, Ni–Fe–B catalysts were facile synthesized by a galvanic replacement method. The catalytic activity was proved to be correlated to the length of time of the replacement reactions. When the replacement time was 60 min, the as-prepared Ni–Fe–B-60 catalyst with a hollow nanochain structure presented the largest surface area and the highest catalytic activity toward hydrogen generation. The experimental studies have revealed that the optimized hydrogen generation performance of the Ni–Fe–B-60 can be explained by the structural effect of the hollow nanochain, the electron effect of electron-enriched Ni active sites, and hydrogen spillover on Fe. Additionally, the overall kinetics of the NaBH_4_ hydrolysis reaction catalyzed by the hollow Ni–Fe–B nanochain catalyst was obtained by a series of experiments. The reusability and separation tests for the hollow Ni–Fe–B nanochain catalyst confirmed that most of the materials' original activity was preserved after five consecutive runs. Thus, the hollow Ni–Fe–B nanochain would be promising catalyst for the generation of hydrogen from hydrides of NaBH_4_.

## Conflicts of interest

There are no conflicts to declare.

## Supplementary Material

RA-008-C8RA03848A-s001
