# Supplementary material for: Facile synthesis of a hollow Ni–Fe–B nanochain and its enhanced catalytic activity for hydrogen generation from NaBH4 hydrolysis
Source: RSC Adv. 2018 Jul 19;8(46):25873–80. doi: 10.1039/c8ra03848a (PMC9082892; doi:10.1039/c8ra03848a)
Supplement: RA-008-C8RA03848A-s001 [file RA-008-C8RA03848A-s001.pdf]

Electronic Supplementary Material (ESI) for RSC Advances.
This journal is © The Royal Society of Chemistry 2018

Supplementary information for

Facile synthesis of hollow Ni-Fe-B nanochain and its enhanced catalytic activity for hydrogen generation from NaBH4 hydrolysis

Jie Guo, Yongjiang Hou*, Bo Li

School of Environmental Science and Engineering, Hebei University of Science and Technology, Shi Jiazhuang 050018, China

*Corresponding author. houyongjiang122@163.com

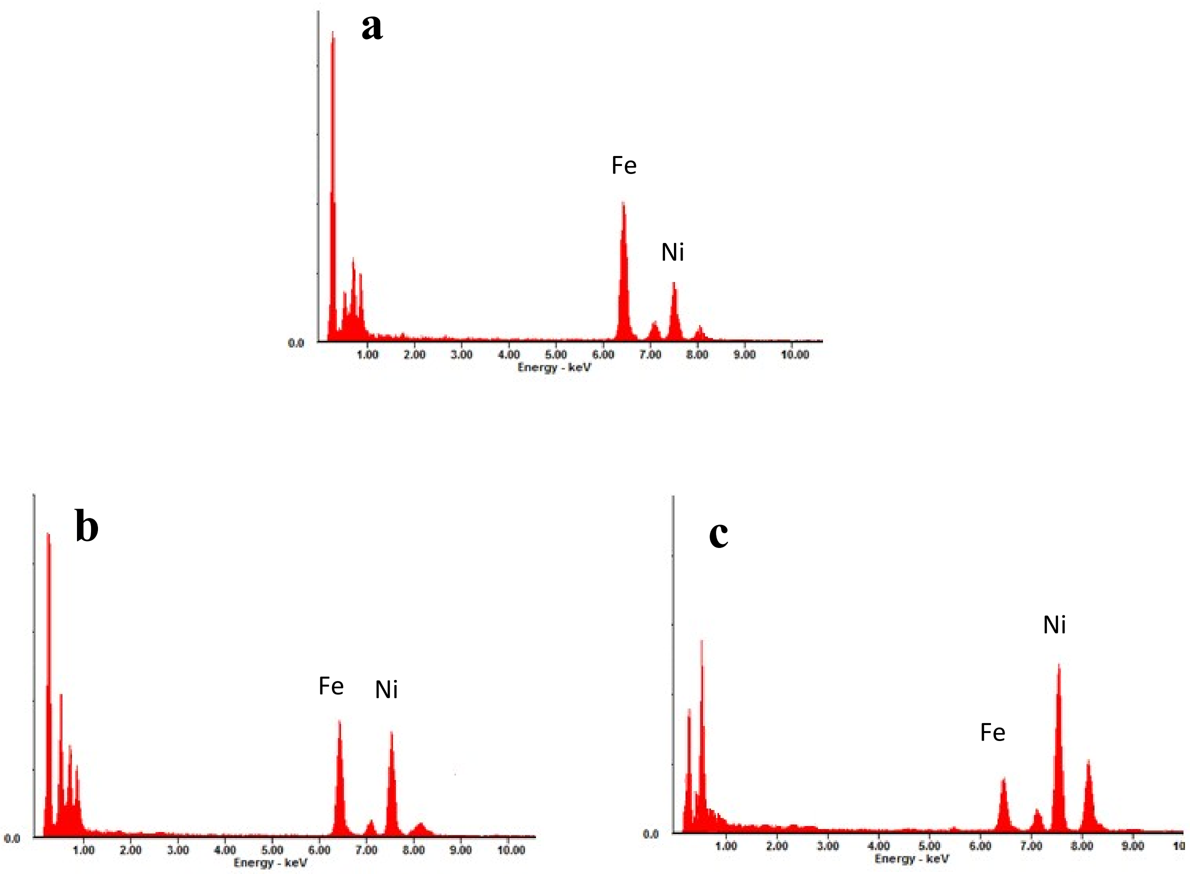

Figure S1 EDX images of (a) Ni–Fe–B-30, (b) Ni–Fe–B-60 and (c)Ni–Fe–B-120 samples.

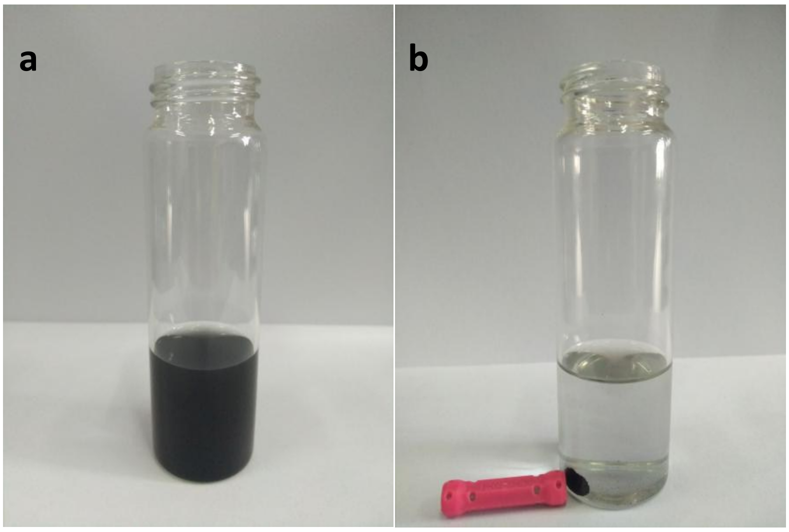

Figure S2 (a) Ni–Fe–B-60 sample dispersed in water, (b) Ni–Fe–B-60 sample effect by the magnet strip.
